# Interaction between Dexamethasone, Ropivacaine, and Contrast Media Used in Interventional Pain Treatment: Considerations in Safety

**DOI:** 10.3390/medicina58121871

**Published:** 2022-12-19

**Authors:** Yeon Ju Kim, Yeon-Dong Kim, Hyungtae Kim, Dong Ji Ahn, Ha-Jung Kim, Won Uk Koh, Young-Jin Ro

**Affiliations:** 1 Department of Anesthesiology and Pain Medicine, Asan Medical Center, University of Ulsan College of Medicine, Seoul 05505, Republic of Korea; 2 Department of Anesthesiology and Pain Medicine, Wonkwang University School of Medicine, 460 Iksan-daero, Iksan 54538, Republic of Korea; 3 Jesaeng-Euise Clinical Anatomy Center, Wonkwang University School of Medicine, 460 Iksan-daero, Iksan 54538, Republic of Korea

**Keywords:** injections, epidural, particulate matter, ropivacaine, steroids, contrast media, pain management, safety

## Abstract

*Background and Objectives*: Although epidural steroid injections are used as an effective treatment, this technique is associated with rare but serious ischemic complications, especially when particulate steroids are used. However, recent studies have reported that even if non-particulate steroids are used, particulates are formed by the interaction with some local anesthetics (LA), causing ischemic complications. This observational study evaluated commonly used combinations of non-particulate steroids and LA with contrast media via microscopic analysis and analyzed the chemical properties of each mixture to identify the correlation of particulate formation. *Materials and Methods*: Commonly used clinical non-particulate and particulate steroids, contrast media, and LA agent combinations were evaluated macroscopically and microscopically. The pH values were also measured at both room temperature (26 °C) and body temperature (36 °C). Where particulates were observed, the particulate size was measured. *Results*: Macroscopically, the mixture of non-particulate steroid and ropivacaine had a slightly cloudy appearance at all concentrations, but there was no visible particulate. However, when observed under a microscope, the pH-dependent particulate formation was observed at all concentration combinations tested. (0.1% ropivacaine: from 19 μm to 70 μm, and 0.2% ropivacaine: from 37 μm to 108 μm at room temperature (26 °C)). When contrast media was mixed or the temperature was raised to body temperature (36 °C), the number and size of the particulates decreased or dissolved. *Conclusions*: The combination of ropivacaine and dexamethasone, a non-particulate steroid, mainly used in epidural injections, forms particulates. However, when mixed with contrast media, particulates are dissolved because of changes in pH and factors affecting particulate formation. In fluoroscopy-guided injections, the use of contrast media could resolve particulate formation.

## 1. Introduction

Epidural steroid injections have become an integral part of pain management for patients with lumbar spinal pain syndromes [1,2,3,4]. Typically, three different routes have been utilized to access the epidural space, including the caudal, interlaminar, and transforaminal approaches. Transforaminal epidural injections have been used to treat lumbar radicular pain since it was first described in 1952 [5], and since then, cervical and thoracic transforaminal epidural injections have also been performed, although rarely, accounting for less than 5% of all cases [6]. Their application is based on the premise that the corticosteroid delivered into the ventral epidural space attains higher local concentrations over an inflamed nerve root, which is considered the main source of pain [7,8].

However, this injection technique is associated with rare but serious ischemic neurologic complications, including blindness, paralysis, and even death [9,10]. Several mechanisms have been hypothesized as the cause of these complications. An infarction of the brain or spinal cord may occur, owing to the injection of particulate steroids into arterial vessels, needle-induced vasospasm, and compression from an epidural hematoma or abscess [11,12,13]. Therefore, these neurologic complications may be related to any other procedure performed in close proximity to the arterial supply of neural structures where inadvertent arterial injection may occur in addition to the transforaminal technique. Compared to other causes, infarction due to the use of particulate steroids can be reduced using a non-particulate injectate. Previous studies reported that particulate steroids have a substantial risk of causing infarction by embolization if inadvertently injected intraarterially compared to non-particulate steroids [14]. 

However, even when non-particulate steroids are used, recent studies have reported that particulates are formed by interactions with some local anesthetics (LA) [15]. For this reason, even if non-particulate steroids are used as the injected drugs, there is a risk of causing complications such as embolic infarction. In actual clinical settings, several LA and steroids are used in various environments; therefore, it is necessary to study the formation of particulates in these situations. Thus, in this observational study, we evaluated the commonly used combination of non-particulate steroids and LA with contrast media via microscopic analysis. In addition, we analyzed the chemical properties of each mixture to find out their correlation with particulate formation.

## 2. Materials and Methods

Commonly used combinations of clinical non-particulate and particulate steroids with contrast media and LA agents were assessed. The analyzed non-particulate steroid was 10 mg/mL dexamethasone sodium phosphate (preservative-free solution; APP Pharmaceuticals, LLC, Lake Zurich, IL, USA), and the particulate steroid was 40 mg/mL triamcinolone. The LA agents examined were 0.1% and 0.2% ropivacaine (preservative-free solution; Naropin^®^, Astra Zeneca, Wedel, Germany). The contrast media used was iopamidol 300 (Pamiray^®^; Dongkook Pharm., Seoul, Republic of Korea), an iodinated nonionic water-soluble contrast agent.

Four mixed samples were prepared for analysis at room temperature (26 °C) and body temperature (36 °C) as follows:

1. 0.1% ropivacaine + dexamethasone,
2. 0.1% ropivacaine + dexamethasone + contrast media,
3. 0.2% ropivacaine + dexamethasone,
4. 0.2% ropivacaine + dexamethasone + contrast media.

A mixture ratio of 1:1 or 1:1:1 was used for the prepared samples and the volume of mixture was 1 ml each.

### 2.1. Macroscopic and Microscopic Analysis of Mixtures

The dexamethasone, 0.1% and 0.2% ropivacaine, and contrast media used in the experiment were each independently observed macroscopically and microscopically. All samples were prepared immediately before imaging. For macroscopic analysis, each mixture was injected into a syringe and observed visually. The samples were also examined with a light microscope (BX 50; Olympus, Tokyo, Japan). For imaging, each of the steroid and LA mixtures was placed on a glass slide over an area of approximately 1 cm in diameter via a standard 25-gauge needle, and a coverslip was applied. Each slide was visually observed after 10 min and examined 5 times each by light microscopy at three magnifications (40×, 150×, and 400×).

### 2.2. Measurement of pH

The default pH values of ropivacaine (0.1% and 0.2%), dexamethasone, and contrast media were measured using an electronic pH probe (Fisher Scientific Accumet AB15 Basic pH Meter, Pittsburgh, PA, USA). The pH measurement of each mixture was additionally analyzed 5 times each at room temperature (26 °C) and body temperature (36 °C), and the value of the measurements was averaged. After mixing each solution, it was observed 10 min later, and the average value obtained from five repeated measurements was used.

## 3. Results

### 3.1. Macroscopic and Microscopic Analysis of Mixtures

**Figure 1:**
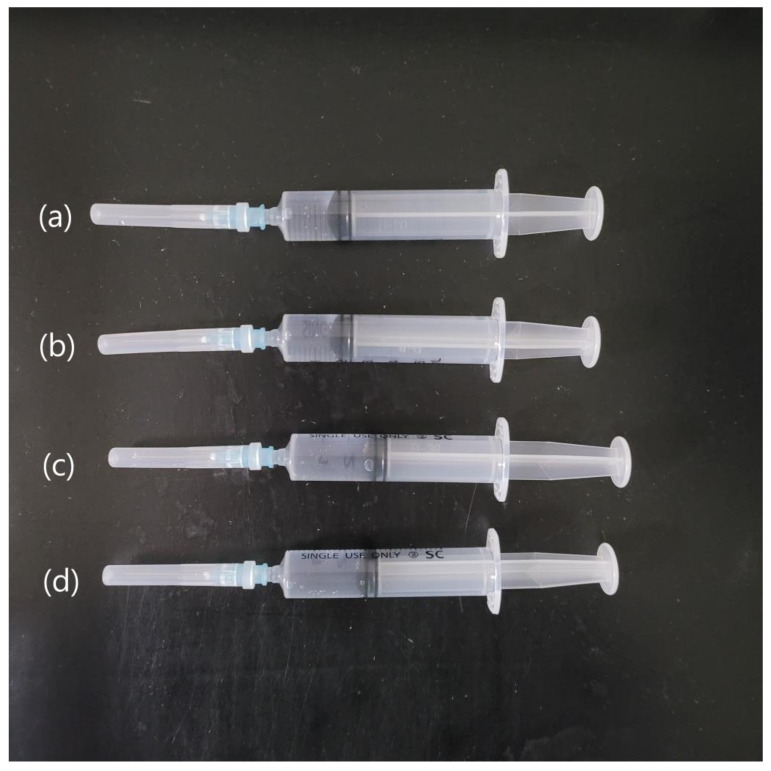
Macroscopic appearance of dexamethasone, ropivacaine, and contrast media. Macroscopically, the mixture of non-particulate steroid and ropivacaine had a slightly cloudy appearance at all concentration. However, there were no visible particulates (**a**,**b**). When contrast media was mixed, it was difficult to visually confirm the difference (**c**,**d**). (**a**) Dexamethasone with 0.1% ropivacaine, (**b**) dexamethasone with 0.2% ropivacaine, (**c**) dexamethasone with 0.1% ropivacaine and contrast media, (**d**) dexamethasone with 0.2% ropivacaine and contrast media.

No identifiable particulates were found in the unmixed solutions of ropivacaine, dexamethasone, and contrast media. Macroscopically, the mixture of non-particulate steroid and ropivacaine had a slightly cloudy appearance at all concentrations. However, there were no visible particulates. In addition, even when the contras media was mixed, it was difficult to visually confirm the difference (Figure 1).

**Figure 2:**
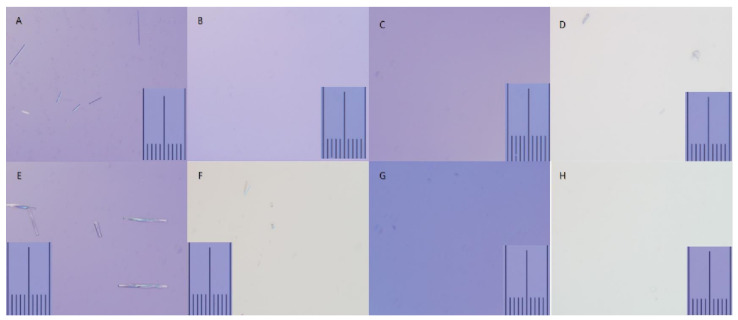
Microscopic findings of dexamethasone, ropivacaine, and contrast media. When ropivacaine was mixed with dexamethasone, particulates were formed (**A**,**E**). When the contrast media was mixed with the mixture or the temperature was increased to 36 °C, particulates were reduced or dissolved (**B**–**D**,**F**–**H**). (**A**) Dexamethasone with 0.1% ropivacaine at 26 °C (400×), (**B**) dexamethasone with 0.1% ropivacaine at 36 °C (400×), (**C**) dexamethasone with 0.1% ropivacaine and contrast media at 26 °C (400×), (**D**) dexamethasone with 0.1% ropivacaine and contrast media at 36 °C (400×), (**E**) dexamethasone with 0.2% ropivacaine at 26 °C (400×), (**F**) dexamethasone with 0.2% ropivacaine at 36 °C (400×), (**G**) dexamethasone with 0.2% ropivacaine and contrast media at 26 °C (400×), (**H**) dexamethasone with 0.2% ropivacaine and contrast media at 36 °C (400×).

When observed with light microscopy, the mixtures of steroid preparations and LA exhibited different characteristics in particulate size and shape depending on the concentration of the injectate, presence of contrast media, and temperature (Table 1, Figure 2). In a previous study, the size of red blood cells was found to be 7.5–7.8 μm in diameter; therefore, this was compared with the size of the particulates formed [16]. 

At 400× magnification, the mixture of 0.1% ropivacaine and non-particulate dexamethasone contained particulates measuring between 19 μm and 70 μm at room temperature (26 °C). The mixture contained long, rod-shaped particulates of varying sizes (Figure 2A). After adding contrast media to the mixture of 0.1% ropivacaine and dexamethasone or when heated to 36 °C, the particulate decreased or dissolved (Figure 2B–D). 

The 0.2% ropivacaine and dexamethasone mixture resulted in several more and larger particulates than at the lower concentration of ropivacaine. When viewed at 400× magnification at room temperature, the particulate size ranged from 37 μm to 108 μm (Figure 2E). In this mixture, when the temperature was increased to 36 °C, the number and size of the particulates decreased but the particulates remained (Figure 2F). When contrast media was added to the mixture at room temperature, all particulates disappeared (Figure 2G). In addition, when mixing contrast media and heating to 36 °C, all the particulates disappeared, and a pure liquid mixture was observed (Figure 2H).

### 3.2. pH Values

The pH of 0.1% ropivacaine in isolation was pH 5.67 at room temperature and pH 5.42 at body temperature. The pH of 0.2% ropivacaine was 5.52 and 5.37 (at 26 °C and 36 °C), respectively, and the pH became more acidic as the concentration increased. Dexamethasone was alkaline, with a pH of 7.76. The pH of the mixture became more acidic when heated, when the concentration of LA increased, and when contrast media was added (Table 2).

## 4. Discussion

The aim of this study was to evaluate the possibility of particulate formation in an environment similar to that in actual clinical practice. Particulate changes were observed depending on the presence of contrast media that was used together in this mixture. In addition, we investigated the pH changes according to the combinations of the solution. Our study found that the 0.1% ropivacaine and dexamethasone mixture formed more particulates at a higher pH. When heated to 36 °C or contrast media was added, particulates disappeared as the mixture’s pH became lower. In the 0.2% ropivacaine and dexamethasone mixture, even when heated to 36 °C, particulates of clinically meaningful size remained and were dissolved only when contrast media was added.

By chemical properties, LA are weak bases which have a pKa close to the normal extracellular pH of 7.4. The pH of commercial LA has to be lower to maximize their water solubility and to increase shelf-life [17]. In our results, a mixture of acidic LA, ropivacaine, and dexamethasone became alkalinized (pH 7.76), and particulates were observed. These results are in agreement with previous studies that demonstrated that alkalinization of LA solutions with sodium bicarbonate causes formation of particulates [18]. This phenomenon is due to the increased proportion of the non-ionized form of the drug when LA is alkalinized, and this non-ionized form tends to be relatively insoluble in water [19]. We observed particulates at different but commonly used clinical concentrations of ropivacaine. The size and number of particulates increased in the 0.2% ropivacaine mixture compared to the 0.1% mixture. These results are supported by the fact that the pKa and changed pH of the LA determine the amount of each agent presented in the basic or protonated form [20], and the concentration of the LA determines the absolute amount of each agent. In addition, this is consistent with a previous study which reported that ropivacaine at higher concentration (0.75% and 1%) forms particulates even at pH 6.0 or higher [21]. Consequently, the higher the LA concentration, the more attention should be paid to particulate formation.

Because the characteristics of particulates generated in the mixture can be changed in vivo, we heated the mixture to 36 °C, which is similar to body temperature. In our study, the number of particulates decreased in both the 0.1% and 0.2% ropivacaine mixtures when heated. This is in contrast to a previous study which found that particulates formed by alkalinization of LA increased as the temperature increased [22]. The mechanism by which a heated mixture shows a decrease in particulates is unclear. However, this discrepancy appears to have been caused by many factors, such as the type and concentration of LA and the magnitude of pH change. In addition, various factors affecting the rate and mechanism of particulates formation from liquid solution include thermodynamics (e.g., solubility, solvent activity, temperature), kinetics (supersaturation, molecular mobility), and molecular recognition (molecular network) [23]. It is thought that the particulate size decreased or dissolved owing to the influence of these other factors in addition to temperature. We also tested the addition of contrast media to commonly used clinical mixtures of ropivacaine and dexamethasone. In our study, the contrast media was weakly acidic (pH 7.0) at room temperature and more acidic (pH 6.64) at body temperature. As can be seen from a previous study where particulates were dissolved when hydrochloric acid (strong acid) was added, it is inferred that particulates dissolve at a certain acidity [24]. Another hypothesis for dissolution proposes the properties of the contrast media. It may be theorized that the dissolving process after the particulate is formed can also be explained by the solubility improving and the stability at near physiologic pH. In the case of cyclodextrin, there is also a study wherein the particulates of alkalized LA were dissolved by increasing the solubility of LA while forming an inclusion complex with LA [25]. However, when these mixtures are injected into the body, it is not certain whether the particulates would dissolve as they would in vitro.

A rare but significant risk of neurologic injury has been reported following the cervical and lumbar epidural injection of corticosteroids [9,26,27]. Multiple mechanisms of neurologic injury have been hypothesized. Previous studies have reported that an infarction of either the brain or the spinal cord, secondary to spasm, trauma, or compression of arteries, can be one mechanism [28,29]. Other potential mechanisms include an embolic mechanism through the inadvertent intra-arterial injection of particulate corticosteroids [12,30]. However, no adverse neurologic complications have been cited when non-particulate steroids have been injected. This is presumed to be because dexamethasone presents particulates significantly smaller than red blood cells, has the least tendency to aggregate, and has the lowest density compared to particulate steroids [16]. However, according to the results of our study, a mixture of non-particulate steroids and LA used in most clinical settings forms particulates larger than the size of red blood cells, increasing the risk of embolic infarct when intra-arterial injection occurs. In addition, even small particulates can occlude the arterioles and capillaries, causing tissue damage. Moreover, ischemic events can occur independent of the injectate mixture as well due to mechanical damage to the arterial supply, including dissection.

Based on our study results, there are certain aspects to be considered in the clinical setting. LA and corticosteroids are commonly administered in combination in clinical practice for various diagnostic and therapeutic purposes [31,32]. The role of corticosteroids in epidural injections to eliminate inflammatory mediators and alleviate damage to nerve fibers [33]. Although the safety of using non-particulate steroids has been experimentally proven, caution should always be exercised in clinical use as the risk of particulate formation when non-particulate and LA are mixed was confirmed again in our study as well as by previous studies [24]. In addition, in the mixtures of LA and non-particulate steroids at concentrations mainly used in clinical practice, particulates are present even if they are not clearly visible macroscopically. Therefore, physicians need to consider the risk of particulate formation via the interaction with LA even when using non-particulate steroids. Another interesting aspect in our results is that particulates were dissolved when the contrast media was mixed in or when the mixture was heated to 36 °C (body temperature). In fluoroscopy-guided injection, simultaneous mixing of LA and non-particulate steroid with contrast media will reduce embolic risk and lower the likelihood of neurologic complications. Further research is needed to compare the changes in more diverse environments and their clinical effects.

There are several limitations of this study. First, the combinations of LA, corticosteroids, and contrast media were observed only in vitro under a microscope. However, in our study, the mixtures were heated to match that of body temperature to make the state as similar as possible to the in vivo environment. Second, the concentration of LA used for our experiment was limited to concentrations mainly used in clinical practice. However, studies on more diverse types of LA and varying concentrations are needed to obtain more detailed information regarding changes in particulate formation. Finally, a clear mechanism as to why particulates are dissolved when the temperature was increased or when contrast media was added has not been elucidated. Further investigation is needed to clarify the exact cause.

## 5. Conclusions

The combination of ropivacaine and dexamethasone, a non-particulate steroid mainly used in epidural injections, forms particulates. However, mixed with contrast media, particulates are dissolved, owing to changes in pH and likely other factors affecting particulate formation. In fluoroscopy-guided injection, the use of contrast media could reduce particulate formation, suggesting the ability to reduce embolic risk. Studies of particulate formation in steroid and LA mixtures and its more detailed clinical effects, particularly in vivo, are still required.

## Figures and Tables

**Table 1:** Subjective microscopic assessment of the particulates.

	Room Temperature (at 26 °C)	Body Temperature (at 36 °C)	With/Add Contrast Media at 26 °C	With/Add Contrast Media 36 °C
0.1% ropivacaine with dexamethasone	++++	clear	clear	+
0.2% ropivacaine with dexamethasone	+++++	+	clear	clear

Extent of particulate ranging from + (minimal) to +++++ (heavy).

**Table 2:** Measurement of pH in the mixed solutions (ropivacaine, dexamethasone, contrast media).

Mixed Solutions	pH
0.1% ropivacaine 1cc + dexamethasone 1cc (26 °C)	7.51
0.1% ropivacaine 1cc + dexamethasone 1cc (36 °C)	7.42
0.1% ropivacaine 1cc + dexamethasone 1cc + contrast media 1cc (26 °C)	7.44
0.1% ropivacaine 1cc + dexamethasone 1cc + contrast media 1cc (36 °C)	7.28
0.2% ropivacaine 1cc + dexamethasone 1cc (26 °C)	7.42
0.2% ropivacaine 1cc + dexamethasone 1cc (36 °C)	7.14
0.2% ropivacaine 1cc + dexamethasone 1cc + contrast media 1cc (26 °C)	7.36
0.2% ropivacaine 1cc + dexamethasone 1cc + contrast media 1cc (36 °C)	7.22

## Data Availability

Not applicable.
